# Post-operative pain assessment, management compliance with WHO guidelines and its barriers in hospitals of West Shoa zone, central of Ethiopia, 2021

**DOI:** 10.1016/j.amsu.2022.104901

**Published:** 2022-11-24

**Authors:** Merga Haile Temesgen, Adamu Brihanu, Zenebe bekele Teshome

**Affiliations:** a Department of Anesthesia, College of Medicine and Health Sciences, Ambo University, Ethiopia; b Department of Psychiatry, College Of Medicine and Health Sciences, Ambo, Ethiopia

**Keywords:** Ambo university, Pain assessment, West Shoa zone, AURH, Ambo University Referral Hospital, BSC, Bachelor Of Science, EPSA, Ethiopian Pharmaceutical Supply Agency, FDG, Focal Group Discussion, NSAID, Non Steriodal Ant iinflamatory Drug, WHO, World Health Organization

## Abstract

**Background:**

Post-Surgical pain should be consistently assessed and documented as vital signs as well as has to be better communicated and adequately managed accordingly. However, there is a limited study regarding pain assessment and management documentation in Ethiopia.

**Objective:**

This study aimed to determine pain assessment documentation, pain management compliance with WHO guidelines, and its barrier.

**Method:**

A cross-sectional retrospective study design mixed with quantitative and qualitative study types was employed. Three hundred sixty-five Patient cards were reviewed from four public hospitals in the West Shoa zone; Central Ethiopia, as well as four key informants groups, were interviewed. WHO guideline was used to review the patient card and a semi-structured questionnaire was used to interview the key informants. Descriptive statistics were used to describe the socio-demographic characteristics; and pain characteristics, and texts, tables, and graphs were used to present the results. Data were analyzed using SPSS-20 and Data from the key informants was thematically analyzed.

**Results:**

From the total of 365 patient cards reviewed, it was observed that only for 189(51.8%) cases pain assessment was done within 48 h after Surgery. Out of the patient who had got pain assessment within 48 h the location of pain was explained in 93(25.5%) cases, pain quality was assessed in 128(35.1%) cases, and pain intensity tool was used in 169(46.3%) cases. Weak opioid (tramadol) is the most commonly described followed by Non-steroid anti-inflammatory drugs to relieve pain after surgery. Only 16(4.4%), patient card side effect was documented and the most reported side effect was Nausea and vomiting (13(3.6%). Lack of regular clinical audits for pain management, lack of technical updates on pain assessment and management as well as knowledge and attitude of health professionals toward pain management were the major barrier to effective pain management.

**Conclusion:**

and recommendation: The pain assessment and documentation in the present study were slightly lower than in previous studies. There was a lack of clinical audit for pain management, a lack of refreshment/technical updates on pain assessment and management, and a lack of regulation of procurement for anti-pain medications. We recommend providing regular technical updates for health professionals and conducting a frequent clinical audit on pain management as well as a designing mechanism for easy availability of anti-paint medications, particularly strong opioids.

## Highlights

• Pain assessment and documentation in this study were lower.
• There was lack of clinical audit and feedback on pain management.
• Lack of continuous supply of anti-pain medications, Health professionals' attitude and knowledge were the main barriers for effective pain management.
• There was lack of technical update on pain assessment and management

## 1 Introduction

Pain is, a subjective phenomenon, defined as an unpleasant sensory and emotional experience associated with actual or potential tissue damage. It can be, classified based on its time course as, either acute or chronic. Acute pain has an abrupt onset and may last up to 6 months if poorly managed [1,2]. Given, the subjectivity of pain, the gold standard for its assessment is a validated self-report tool. Where self-report is not possible, such as with communication difficulties, behavioural assessment tools assessing vocalization, facial grimacing, and restlessness are indicated [3,4]. The most commonly used self-report tools for evaluating pain intensity in an acute setting are the Likert-type numeric rating and the visual analog scales [5]. The pain tool selected should be used regularly to assess pain and the effect of interventions. It should not, however, be used as the sole measure of pain perception. Location, quality of pain, and current aggravating and alleviating factors are additional assessment elements useful in selecting interventions to manage pain [[6], [7], [8]].

Adequate documentation of pain management and regular pain assessment is essential to achieve sufficient pain relief after surgery. Documentation can support continuity of care and provides an important means of communication between clinicians, Consistent documentation provides legal evidence of the caring process and supports evaluation of the quality of care [8,9].The time frame for reassessment also should be directed by hospital or unit policies and procedures. The goal of acute pain management is to prevent postoperative complications, speed up healing, minimize side effects caused by analgesics, prevent acute pain from becoming chronic pain, and reduce the frequency and severity of pain [[10], [11], [12]]. An example of a multimodal analgesia approach is the World Health Organization (WHO) analgesic ladder. In this conceptual ladder, analgesics are adjusted in a stepwise manner from non-opioids through to potent opioids (step-up) or vice-versa (step-down), consistent with the patient's reported pain intensity [13].

Several barriers (system-related, staff-related, nurse-related, physician-related, and patient-related) have been identified that hinder health care professionals from achieving optimal pain management [14,15]. System-related barriers include a lack of clearly defined standards and pain management protocols and limited access to pain specialists and analgesics [16], Staff-related barriers include inadequate knowledge and skills, lack of teamwork, Lack of knowledge, and false concerns about addiction and overdosing are examples of physician-related barriers, heavy workload, and lack of time [17,18], Reluctance to take analgesics, fear of side effects and fear of addiction are examples of patient-related factors [19,20]. The annual surgical volume is increasing throughout the world. In the year 2012 alone, 266.2 to 359.5 million operations were performed, representing a 38% increase over the previous eight years; in low-income countries, there also is an estimated high proportion of surgical volumes due increase in emergency obstetric procedures [[21], [22], [23]]. The rise in the number of operations is not without risk; nearly 80% of patient experiences pain after surgery of which 78–86% have moderate, severe, or extreme pain [24].

Poorly managed postoperative pain can impair surgical outcomes, it may become chronic pain [25,26], it can result in a prolonged duration of the hospital stay to more severe complications [27], atelectasis, respiratory infection, myocardial infarction, and even bad surgical outcome, death [28,29] and overall it increases costs for society [30]. Adequate pain management is one of the most important factors in expediting recovery, by enabling fast mobilization [31]. Although pain management continues to be a problem in both developed and developing countries, sadly the suffering from untreated pain is larger and more troublesome among economically disadvantaged individuals [32,33]. Therefore our study aimed to determine pain assessment documentation, pain management compliance with WHO guidelines; and its barrier.

## 2 Methods

### 2.1 Study setting and period

In West Shoa Zone there are nine Public hospitals. Among these one referral hospital and three General hospitals namely, Ambo University Referral Hospital (AURH), Ambo General, Gedo, and Ginda berat purposefully selected. The study was conducted from January to February 2021. The study was registered in the research registry UIN = 8227 and the manuscript was Witten according to STROCESS guidelines [34].

### 2.2 Study design

Both quantitative and qualitative study methods were employed Retrospective Quantitative Cross-sectional study, mixed with a Qualitative study type was applied to collect data.

### 2.3 Sample size and sampling technique

#### 2.3.1 Sampling technique

**Fig. 1:**
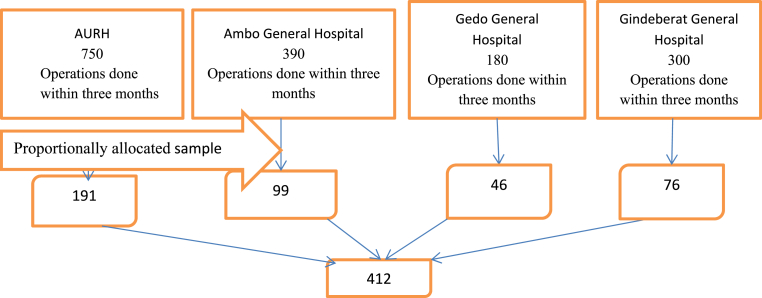
Schematic distribution sample size for the selected hospitals.

A convenient sampling technique was used to select hospitals. A simple Random sampling method was used to select cards that full fill inclusion criteria. The total number of patient cards for each hospital that had surgical procedures from January 01 up to March 30/2021 was determined. Then the total sample size was allocated proportionally to each selected hospital based on the target population. Finally, the patient cards were selected from the operation register by lottery method from each hospital until desired sample size fully filled (Fig. 1).

### 2.4 Inclusion criteria

Patient who was hospitalized for 48 h after surgery.

### 2.5 Variables

#### 2.5.1 Dependent variables

• Post-Operative Pain assessment and its management

#### 2.5.2 Independent variables

• Socio-demographic characteristics of Nurse (Age, sex, Educational status, work experience)
• Barriers to effective pain documentation and management (system-related (lack of clearly defined standards and pain management protocols, and access to analgesics), health professionals related (inadequate knowledge and skills, lack of teamwork, and heavy workload.

### 2.6 Operational definitions

**Post-operative pain**: Pain present in a surgical patient for 48 h after a procedure.

**Pain assessment:** Refers to the documentation of pain location, description of pain character, and measure of pain intensity with the use of an assessment tool.

Pain intensity was categorized according to the following measurement scale:

**No pain** level of 0 on the numerical pain scale.

**Mild Pain**: level of pain on a numerical pain scale between 1 and 3.

**Moderate pain**: level of pain on the numerical pain scale between 4 and 6.

**Severe Pain**: level of pain on the numerical pain scale ranging from 7 to 10.

### 2.7 Data collection tool and data collectors

The checklist was prepared from WHO pain management guidelines and pain management guidelines for low-resource setting countries. The checklist consists of age, gender, admission ward, type of procedure, Pain intensity, Location, number of pain assessments recorded, an assessment tool used, type of analgesics used, route of administration, and number of any reassessment conducted.

A semi-structured questionnaire was prepared to lead FGD to dig out barriers to effective pain management. Eight BSc nurses were recruited as data collectors and three BSC people with health-related backgrounds supervised the data collection process.

### 2.8 Ethical clearance

Ethical clearance was obtained from the college of medicine and health science ethical clearance committee before the start of the study and official support letter was written to responsible personnel to each hospital to gate permission for data collection. Confidentiality was preserved at all levels of the study by avoiding personal identifiers and using codes to identify patients.

## 3 Result

### 3.1 General characteristics of study population among public hospitals (N = 365)

From the total of 412 patient card considered to be reviewed only 365 cards fulfill the inclusion criteria which make the response rate 88.6%.The main reason for discarding the patient card was data incompleteness. Of these 365 cards, 224(61.4%) cases received General anesthesia and 194(53.2%) of them underwent Emergency surgery. Cesarean delivery 133(36.4%) is the most commonly performed surgical procedure followed by laparotomy 96(26.3% (Table 1).

**Table 1:** General characteristics of patient underwent surgical procedure at Public hospital.

Variable		Frequency	Percentage
Types of ward	General surgery	197	54.0
	Oby and gyn ward	168	46.0
Type of Anesthesia	General	224	61.4
	Regional	141	38.6
Type of surgery	Emergency	194	53.2
	Elective	171	46.8
Surgical procedure done	Cesarean delivery	133	36.4
	Mastectomy	11	3.0
	Laparotomy	96	26.3
	Thyroidectomy	24	6.6
	Myomectomy	16	4.4
	Hysterectomy	13	3.6
	Prostectomy	9	2.5
	Hernioraphy	12	3.3
	*others	51	14.0

### 3.2 Pain assessment and management

**Fig. 2:**
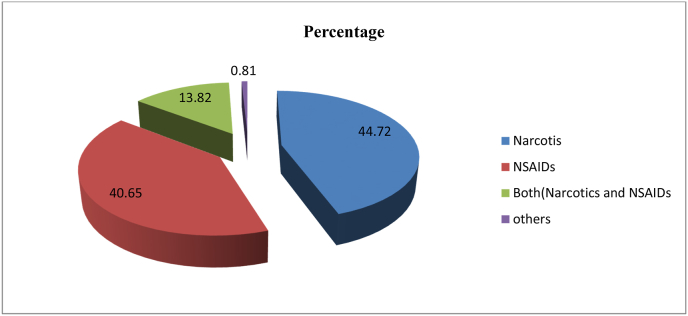
Type of Anti pain given within 48 h after surgery among public Hospitals N = 365).

From the total of 365 patient cards reviewed pain assessment was done only for 189(51.8%) cases within 48 h after Surgery. Among those cards in; 72(19.7%) of them the assessment was done twice whereas for 46(12.6%) it was done three times. Out of the patient who had got pain assessment within 48 h the location of pain was explained in 93(25.5%) of the cases, pain quality was assessed in 128(35.1%) of cases, pain intensity tool was used in 169(46.3%). Regarding the type of pain assessment scale, the most commonly used scale was verbal scale 136(37.3%) followed by Numeric 24(6.6%) (Table 2 and Table 3), (Fig. 2).

**Table 2:** Description of pain Assessment and Reassessment among patient underwent surgical procedure at Public hospital.

Variables		Frequency	Percentage
Pain assessment with 48 h	Yes	**189**	**51.8**
	No	176	48.2
Frequency pain assessment	Once	30	8.2
	Twice	71	19.5
	Three times	45	12.3
	Four times	18	4.9
	Five times	8	2.2
	nine times	5	1.4
	ten and above	2	.5
Location Assessment	Yes	93	25.5
	No	272	74.5
Pain quality assessment	Yes	128	35.1
	NO	237	64.9
Utilization of pain intensity tools	Yes	169	46.3
	No	196	53.7
Type of pain scale used	Visual	9	2.5
	Numeric	24	6.6
	verbal	136	37.3

**Table 3:** Opoid given within 48 h after surgery among four selected Hospitals.

Variables		Frequency	Percentage
Opoid Given	Yes	163	44.7
	NO	202	55.3
Type of opoid given within48 h	Weak	134	36.7
	Strong	29	7.9

**Table 4:** Anti-pain Side effect documentation and its management among four selected Hospitals (N = 365).

Variables		Frequency	Percentage
Documented side effect	Yes	16	4.4
	NO	349	95.6
Type of Side effect	N/V	13	81.25
	Itching	1	6.25
	Respiratory depression	2	12.5
Treatment for side effect	Yes	7	43.75
	No	9	56.25
Type of drug given for side effect	Others(oxygen, cimetidine, methochlopromide)	6	37.5
	Not indicated	9	62.5

### 3.3 Anti-pain side effected documented

Only 16(4.4%) documented ant pain side effect and the most reported side effect was Nausea and vomiting (13(3.6%).From Reported/Documented side effects 6 (37.5%) had got treatment (Table4).

### 3.4 Barriers to effective pain assessment and management

#### 3.4.1 Hospital management related barriers

It reported that there is no regulation of procurement of anti-pain drugs except Ethiopian Pharmaceutical Supply Agency (EPSA) which makes the supply of drugs inaccessible when needed. No clinical audit on pain assessment and management, and Less emphasis from administrative bodies to achieve the Federal Ministry of Health plan that needs to consider pain as the fifth vital sign and make pain-free hospitals. The newly recruited health professional don't get refreshment/induction training before starting providing health care services and no sustainable technical update on Pain assessment and management.

#### 3.4.2 Health professional-related barriers

Health Care Professionals’ lack of knowledge, attitude, and skills to effectively halt pain after surgery has been reported for a while. It is largely associated with a lack of continuous supply of adequate analgesics (Narcotics) and the unavailability of approved national pain guidelines.

## 4 Discussion

The findings of this study revealed that from the total of 365 patient cards reviewed pain assessment was done only for 189(51.8%) cases within 48 h after Surgery. Out of the patient who had got pain assessment within 48 h, the location of pain was explained in 93(25.5%) cases, pain quality was assessed in 128(35.1%) cases, and pain intensity assessment tool was used in 169(46.3%). The pain management side effect was documented only for sixteen cards and from these six patients’ treatment given for side effects was documented. Knowledge, attitude, and skill of health care providers, lack of adequate Narcotics, and lack of guidelines as well as less emphasis from administrative bodies to achieve the Federal Ministry of Health plan which considers pain as the fifth vital sign to make pain-free hospitals are the common types of barriers to pain assessment and management.

According to the study done in South Africa on Pain assessment and management nearly half(51%) of the card pain assessment was documented and the Verbal pain assessment scale was the most commonly used [13]. The findings of the present study are almost similar regarding pain assessment and assessment tools used.

The present study reviewed 51.8% of cards in the pain assessment documented and the Verbal pain assessment scale is the most commonly used. This finding is almost similar to Pain assessment and management: An audit of practice at a tertiary hospital [13]. A study done in Iran on the Management and documentation of postoperative pain by nurses indicated that only 5.2% of patient pain quality was assessed and almost all patients with pain locations were documented. On the contrary in the present study, only 35.1% of patients with pain had got a pain quality assessment. This is quite greater than the Iran study [35]. This discrepancy might be due to cultural differences that can be expressed in some cultures describing pain quality as not acceptable as well as Patients usually avoid expressing their pain in the fear of drug side effects or being popular as the bothering client among busy health professionals.

According to Jordan's study, 65% of the pain assessment and management was documented on the patient card and 61% of pain location was described [36]. This is quite greater than the present study findings. The possible reasons for the differences might be Knowledge and attitude of health professionals toward pain assessment and management were a barrier in the present study which might be in Jordan as well as National attention on pain assessment and management can be different.

A study done at Pacific Northwest revealed that 99.6% of paint assessment was documented and valid pain scales (either descriptive or numeric; 99.6%), pain character (88.2%), and pain location (90.4%).were identified [37]. This is much higher than the finding of the present study which might be due to the presence of Institutional pain management policies at the study site that required nurses to conduct pain assessment at least once every 8 h after admission and there is a difference in curriculum and socioeconomic status between the two countries [37]. In our study about 16(4.4%), anti-pain side effects were documented and the most reported side effect was Nausea and vomiting (13(3.6%). This finding is almost similar to the study done in Niger [38].

According to baseline evaluation of pain management practices and teaching health facility and Health technical school; limited availability of pain-relieving drugs and procedure sets to administer.

Drugs, unavailability of guidelines for pain management that health professionals are expected to follow, lack of knowledge about the pain-relieving drugs, mechanisms of pain and management as well as poor attitude of health professionals towards pain and its management were identified as barriers to pain assessment and management which in line with the present study [39].

According to the study done in Niger on the Management of postoperative pain, 76.5% of the patients had used tramadol as pain management, and 12.1% used Non-steroidal anti-inflammatory drugs [40]. While in the current study only36.7% had used weak opioids (tramadol) and 40.65% had used Non-steroidal anti-inflammatory drugs (NSIAD). This difference might be due to fear of the side effects of opioids as well as the availability of opioid medications in our case. NSAIDs are easily available compared to opioids and patients prefer NSAIDs because it's less costly and has fewer side effects.

## 5 Conclusion

The magnitude of pain assessment and management in this study is lower compared to previous similar studies and lack of technical updates, lack of frequent clinical audits, and an inadequate supply of anti-pain, as well as knowledge and attitude of health professionals, were the main barrier for effective pain management.

### 5.1 Recommendation

#### 5.1.1 Hospitals

➢ the hospital management bodies are expected to update the knowledge of health professionals on pain management
➢ Hospitals are expected to ensure Pain management guidelines availability and their utilization
➢ It is better if the hospital conducts a frequent clinical audit on pain management
➢ Design special procurement and stock management system for antipain medications

### 5.2 ➢health professional

➢ Would give attention to documentation of pain assessment and considering pain as five vital signs
➢ Let health professionals consider non-pharmacological therapy for pain management

#### 5.2.1 Researcher

➢ Research is expected to investigate the pain management policy, strategy, plan, and mechanism of anti-pain availability and unseen issues related to pain management using large-scale studies.

### 5.3 Limitation and challenges

One of the limitations of this study could be the use of secondary data (patient card reviewed) and the main challenge was security instability during data collection.

### 5.4 Future implication of the study

As per the recommendation of the study for future, the concerned bodies should hard work in order to implement pain management as fifth vital sign as well as further study should be done to evaluate the policy of the country to check if it supports the initiative of provision of pain free hospitals considering of the pain management as fifth vital sign.

## Funding information

This research was funded by 10.13039/501100016392Ambo University.

## Ethical approval

Ethical approval was secured from Ambo University institutional review board.

## Please state any sources of funding for your research

Fully funded by 10.13039/501100016392Ambo University.

## Author contribution

Merga Haile, Adamu Birhanu and Zenebe Bekele have made substantial contributions to conception, Writing - review and also contributed in editing of the manuscript drafts for scientific merit and depth.

## Consent

No individual and sensitive data and Data are available on request.

## Registration of research studies

1.Name of the registry: Researchregistry.com
2.Unique identifying number or registration ID: researchregistry8227
3 … \merga manuscript\Register Now - Research Registry merga.pdf

## Guarantor

Zenebe bekele and Merga Haile.

## Provenance and preview

Not commissioned, externally peer reviewed.

## Declaration of competing interest

The authors have no competing interests to declare.
